# A serial mediation model of negative life events on school adjustment of left-behind adolescents in rural china: the central role of hope and gratitude

**DOI:** 10.1186/s12888-023-05102-2

**Published:** 2023-08-14

**Authors:** Lyuci Zhang, Samsilah Roslan, Zeinab Zaremohzzabieh, Kexin Liu, Xing Tang, Yuqin Jiang, Zulkifli Mohamad

**Affiliations:** 1 Faculty of Educational Studies, Universiti Putra Malaysia, 43400 Serdang, Malaysia; 2 Department of Education and Music, Hezhou University, Hezhou, 542899 China; 3 Institute for Social Science Studies, Universiti Putra Malaysia, 43400 Serdang, Malaysia; 4 Youth League Committee, Guangxi Medical University, Nanning, 530021 China; 5 School of Intelligent Manufacturing, Nanning College For Vocational Technology, Nanning, 530008 China; 6 Faculty of Human Development, Universiti Pendidikan Sultan Idris, Tanjung Malim, 35900 Perak, Malaysia; 7 Pusat Pengajian Citra Universiti, Universiti Kebangsaan Malaysia (UKM), 43600 Bangi, Selangor Malaysia

**Keywords:** Negative life events, Hope, Gratitude, School adjustment, Chinese left-behind adolescents

## Abstract

Adjustment difficulties of school students are common and their school adjustment has gained wide concern in recent years. Negative life events (NLEs) hope, and gratitude have been associated with school adjustment. However, the potential effect of NLEs on hope and gratitude and whether hope and gratitude mediate the association between NLEs and school adjustment among high students have not been studied. Thus, this study aims to investigate the association between NLEs, hope and gratitude, and school adjustment in high school students in China. Additionally, the study aims to examine the mediating role of hope and gratitude in the association between NLEs and school adjustment. A total of 700 junior high school students in Guangxi Province (336 boys, 364 girls, M age = 15 years) completed the questionnaire. The results indicated significant mediating effects of hope and gratitude in the sequential positive association between NLEs and school adjustment. Furthermore, this study unraveled the complexity of the link between NLEs and school adjustment with the combination of hope and gratitude. The findings emphasized the importance of fostering hope and gratitude in left-behind adolescents to combat the negative consequences of NLEs. The study is also one of the first to investigate a serial mediation model to determine which NLEs influence Chinese left-behind adolescents’ school adjustment.

## Introduction

Urbanization has been a significant worldwide issue for centuries. By 2050, 68% of the world's population will reside in cities, with a significant portion of them being Chinese. According to estimates, the population in China will be 255 million by then [1]. China continues to have the fastest rate of urbanization in the developing world and currently has the highest rate of urban population growth globally. By 2050, approximately 20.2% more migrants will live in cities around China than in 2018 (11.3%) [2]. Migration is a significant contributor to urbanization. When a large number of migrants from the agricultural population move to cities, the urbanization process is accelerated [3]. According to China’s National Bureau of Statistics [4], the total number of migrant workers in 2019 was 290.77 million, with 75.08 million migrating across provinces and 99.17 million working within provinces.

Left-behind adolescents in rural China are increasingly common due to the significant increase in migrant workers. As per the national definition of a migrant population, left-behind adolescents are individuals under the age of 18 who have been left-behind at their original residence while one or both parents migrate to cities for work. These adolescents have not lived with one or both parents for at least six months [4]. According to UNICEF China [5], in 2015, there were 40.51 million left-behind adolescents in rural China. Researchers have found that these adolescents are at a crucial stage of mental development and their mental health is often more affected than their physical health [6]. Despite parents' migration to support their education, left-behind adolescents often struggle with school adjustment and are more prone to academic and behavioral issues [7, 8]. Lam and Yeoh [9] suggest that their poor school performance may be attributed to unstable home environments, insufficient parental care, and a lack of mental health education in their schools. Chen et al. [10] discovered that approximately 75% of adolescents with migrant parents performed poorly in school. Additionally, Ezaki [11] found that 473 Nepalese left-behind elementary school students displayed low academic performance.

Multiple studies consistently indicate that negative life events (NLEs) pose significant challenges to school adjustment [12–14]. NLEs encompass adverse or difficult experiences encountered by left-behind adolescents in rural China, such as parental migration. When one or both parents migrate to other cities for work, it often leads to feelings of loneliness, separation anxiety, and negatively impacts the emotional well-being and concentration of these adolescents as they navigate their school environment [13]. School adjustment encompasses various domains, including academic performance, social integration, emotional well-being, and engagement in school-related activities. It reflects the ability of rural Chinese left-behind adolescents to cope with the challenges arising from parental migration, family dynamics, and limited support systems [15].

Moreover, Anders et al. [16] conducted a study involving 1084 undergraduate and community college students, revealing that those who experienced a greater number of life events exhibited poorer outcomes in various aspects of school adjustment. These outcomes included lower grade point averages, increased distress, and diminished life satisfaction. Similarly, Reyes-Rodríguez et al. [17] conducted a survey that indicated that negative life events heightened the prevalence and severity of depression symptoms among freshmen college students, a common emotional struggle during their academic journey. Additionally, ample evidence suggests that negative life events contribute to suicidal behavior and other detrimental outcomes throughout college life [18, 19]. As a result, researchers and educators share a pressing concern to identify strategies that can alleviate the adverse effects of negative life events on school adjustment. It is worth noting that positive psychology acknowledges the existence and impact of negative life events on students' adjustment, but it emphasizes understanding how individuals can effectively navigate and cope with these challenges. This field highlights the importance of promoting positive emotions, strengths, and psychological resources to foster resilience and facilitate adaptive adjustment in the face of adversity.

Previous research has indicated that interventions targeting hope and gratitude can serve as vital psychological resources to promote students’ adjustment [20]. Hope, in this context, is defined as the ability to cultivate a sense of agency and identify pathways toward achieving personal goals [21]. By fostering hope and gratitude, the study seeks to enhance the psychological resilience of left-behind adolescents, providing them with the necessary resources to navigate challenges in school life and mitigate the adverse effects of NLEs. It has been linked to increased subjective well-being, optimism, achievement, self-belief, and psychological well-being [22, 23]. When confronted with hardship, those with high hope may be able to adjust their targets and tap into recourses, while those with low hope illustrate a lack of flexibility through disengagement or negative thinking. In addition, hope was used as a mediating variable to study adjustment in past studies. For example, Stanton et al. [24] proved that the mediation of hope and coping strategies assisted in predicting adjustment among breast cancer patients. Additionally, Akdeniz and Gültekin Ahçı [25] investigated how hope mediated the relationships between loneliness and psychological adjustment issues.

Gratitude is an attitude or disposition that makes a person aware of and appreciative of the good things in life, which causes them to feel good and act prosocially [26]. There is proof that practicing gratitude can help adolescents achieve a range of beneficial outcomes, including higher self-esteem, better social and emotional functioning, and improved academic performance [27, 28]. Specifically, Fredrickson's extended construction theory [29] provides insights into the relationship between gratitude and negative life events. According to the theory, gratitude acts as a positive emotion that can counterbalance the negative impact of adversities and negative life events. When individuals experience negative life events, such as hardships, challenges, or trauma, they may be overwhelmed by negative emotions and thoughts. However, gratitude, as an attitude or disposition, can serve as a psychological resource to help individuals navigate and cope with these negative experiences.

By cultivating gratitude, individuals become more aware of and appreciative of the positive aspects of their lives, even amid adversity. This shift in focus allows them to find meaning, draw strength, and derive a sense of appreciation from their experiences, including negative life events. Gratitude helps individuals reframe their perspective and encourages them to focus on the positive aspects of their circumstances.

In this way, gratitude can mitigate the impact of negative life events by helping individuals maintain a more positive outlook, facilitating their ability to cope, adapt, and recover from adversity. By emphasizing gratitude, Fredrickson's extended construction theory suggests that individuals can find resilience, growth, and positive outcomes even in the face of challenging life events [30]. Similar to hope, gratitude can act as a protective shield against traumatic events by encouraging left-behind adolescents to see the positive aspects of their lives and providing them with a sense of purpose and motivation for their plans. Past research has found a mediating effect of gratitude on adaptability. For example, Bryant et al. [31] identified gratitude as a mediator of the favorable benefits of savoring toward aging. In another previous study, gratitude was a mediator in the association between maternal emotional support and self-esteem [32].

In light of the aforementioned context, this study seeks to address the existing research gap concerning predictors of school adjustment in rural Chinese adolescents. While an increasing body of literature acknowledges the influence of NLEs on positive social adjustment [14, 33], there are still gaps in our knowledge, no research has been conducted on the potential impact of NLEs on gratitude and hope, and whether gratitude and hope mediate the association between NLEs and school adjustment have not been examined in the context of Chinese left-behind adolescents. In light of the aforementioned issues, the current study seeks to: (1) study the association among NLEs, gratitude, hope, and school adjustment, and (2) examine whether the association between NLEs and school adjustment among Chinese left-behind adolescents is mediated by gratitude and hope. Authors hypothesize that Chinese left-behind adolescents who suffer more NLEs will have lower gratitude and hope scores and poorer school adjustment, and that gratitude and hope mediate the association between NLEs and school adjustment as displayed in Ang et al. [20].

The results of the current study will help educators and adolescents who have been left-behind create initiatives that will raise their sense of appreciation and optimism, improve their physical and emotional well-being, and strengthen their ability to adjust to school.

## Current study

**Fig. 1:**
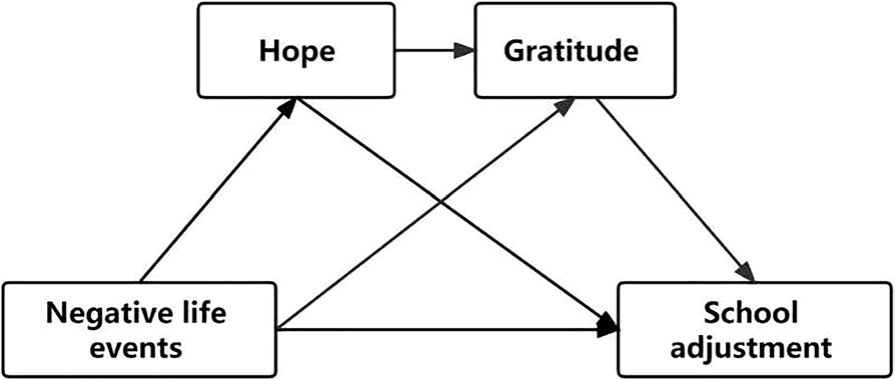
Hypothesized model of the associations between the study’s constructs

A current study has investigated the effect of NLEs on Chinese left-behind adolescents’ school adjustment. Adolescents experience a delicate phase of development marked by exposure to stressful life events. The adjustment of adolescents to school represents a significant societal concern that requires particular attention [34]. By delving into the potential psychological mechanisms underlying the relationship between stressful life events and school adjustment during adolescence, we can potentially diminish the adverse effects of such events on their school adaptation. This exploration holds crucial theoretical and practical significance [35]. No studies, however, have looked at the presence of multiple psychological resources (such as hope and gratitude) and how they interact in reducing the adverse effects of NLEs on school adjustment in samples of Chinese adolescents who were left-behind. Fewer studies [8, 36] have examined how these adolescents adjust to school. The current study aims to comprehend the underlying mechanisms relating NLEs to school adjustment among Chinese left-behind students. This study believes that the association between NLEs and school adjustment may be serially mediated by the two mediators of hope and gratitude. Therefore, NLEs have an incremental effect on school adjustment through hope and gratitude. This study will test this serial mediation hypothesis by conducting a large-scale survey of left-behind adolescents in rural China. The current study offers the following four hypotheses (Fig. 1):

- H_1_: There is a significant negative association between NLEs and school adjustment among Chinese left-behind adolescents.
- H_2_: Hope mediates the association between NLEs and school adjustment among Chinese left-behind adolescents.
- H_3_: Gratitude mediates the relationship between NLEs and school adjustment among Chinese left-behind adolescents.
- H_4_: Hope and gratitude serially mediate the effect of NLEs and school adjustment among Chinese left-behind adolescents.

## Methodology

### Participants and procedures

The participants of this study were Chinese rural adolescents (700) aged 11 to 17 years old (M age = 13.50, SD age = 1.05) and enrolled in grades 7 to 9 from six rural junior high schools in Guangxi Province, China. By separating the population into various subgroups or strata according to certain criteria, stratified cluster sampling includes choosing clusters from each stratum. In this case, the researchers stratified the population of Chinese rural adolescents based on their enrollment in grades 7 to 9 from six rural junior high schools in Guangxi Province. The steps involved in the stratified cluster sampling technique for this study would likely be as follows:

First step: the researchers identified the stratum as rural junior high schools in Guangxi Province. They may have considered factors such as geographical location, school size, or academic performance to ensure the representation of different types of rural schools.

Cluster Selection: From each stratum (each rural junior high school), the researchers randomly selected clusters, which are intact groups or classes of students. The number of clusters selected from each school may vary depending on the research design and desired statistical power.

Second step: Within each selected cluster, the researchers applied the inclusion criteria to identify eligible participants. They would have confirmed that the selected students met the requirements of normal intelligence, effective questionnaire response, and being left-behind adolescents under the age of 18 who have been left-behind in their hometown for more than six months due to parental migration.

Third step: The researchers determined the desired sample size of 700 Chinese rural adolescents based on factors such as feasibility, available resources, and statistical power considerations.

- The researchers would divide the population of Chinese rural adolescents into distinct subgroups based on gender and grade. In this case, the two strata would be "males" and "females" for gender, and "grade 7," "grade 8," and "grade 9" for grade.
- Within each stratum, the researchers would determine the target number of participants to be sampled based on the proportion of each subgroup in the population. They would allocate a proportional number of participants to each subgroup to ensure that the sample reflects the gender and grade distributions observed in the population.

For example, if the target sample size is 700 participants, the researchers would allocate approximately 336 participants (48% of 700) to the male subgroup and 364 participants (52% of 700) to the female subgroup to maintain the same gender distribution. Similarly, they would allocate approximately 238 participants (34% of 700), 234 participants (33.4% of 700), and 228 participants (32.6% of 700) to grade 7, grade 8, and grade 9, respectively, to reflect the grade distribution.

By employing stratification and proportional allocation, the researchers can ensure that the final sample represents the gender and grade distributions observed in the population. This approach allows for controlling these demographic variables, which can help analyze and interpret the results while drawing conclusions about the entire population of Chinese rural adolescents in different genders and grades.

## Measures

### Negative life events

NLEs were measured for this study through the Adolescent Self-Rating Life-Events Checklist [ASLEC; [37]]. ASLEC is a self-rating questionnaire consisting of 27 NLEs that might bring psychophysiological reactions to adolescents. The assessment period might be three months, six months, nine months, or one year, depending on the research purpose. The current Cronbach Alpha reliability coefficient for this scale was 0.920.

### Hope

This measurement is based on Snyder’s [21] initial definition of hope. The Hope Scale consists of six items and has two dimensions: agency thinking and pathway thinking (α = 0.816). The current Cronbach Alpha reliability coefficient for this scale was 0.816.

### Gratitude

The Gratitude Questionnaire-Six-Item Form (GQ-6) is employed to test gratitude [38]. On a seven-point scale, teenagers indicate their level of agreement with each of the six items. Initial research showed that GQ-6 might be appropriate for adolescents [39–41]. The current Cronbach Alpha reliability coefficient for this scale was 0.779.

### School adjustment

This study adopted the School Adjustment Questionnaire for Junior High School Students to measure school adjustment. The School Adjustment Questionnaire for Junior High School Students was developed by Cui (2008) to measure respondents’ perceived level of school adjustment. The questionnaire comprises a total of 27 items. All projects are scored on a five-point Likert scale, including five dimensions: academic adjustment, peer relationship, school attitude and emotion, routine adjustment, and teacher-student relationship among middle school students. The higher the total score, the better the adaptation to school. The current Cronbach Alpha reliability coefficient for this scale was 0.917.

### Procedures

Six middle school classrooms were chosen at random for group testing. All subjects completed the above four questionnaires. The Universiti Putra Malaysia’s ethics committee accepted the study procedure.

### Data analysis

First, an independent sample t-test was used to study the influence of gender on NLEs and school adjustment. Second, an analysis of variance (ANOVA) was employed to compare the differences in grades on NLEs and school adjustment. Third, to evaluate the correlations between all research variables, a Pearson correlation analysis was performed. Finally, a regression-based approach called mediation analysis was developed to examine the proposed mediation models. It was based on correlations between the variables. The macro-program PROCESS 2.1 and SPSS v.26 were employed to analyze the data that had been collected [42].

## Results

### Common method deviation T-Test

Harman’s [43] single-factor analysis was employed in this study to assess the common source variation. Without rotation, an exploratory factor analysis (EFA) was carried out. Based on the evaluation, 13 factors were found with higher values of 1 and the variance explained by the first factor was 19.663%, which was no more than 40%. Therefore, no serious common-method bias was observed between the measured variables in the study [44].

### Descriptive analysis and correlation analysis

Findings from the independent samples t-test revealed significant gender differences in school adjustment and no significant differences in NLEs (*P* = 0.215). The results indicated that female left-behind adolescents’ school adjustment was higher than male left-behind adolescents (*P* = 0.017). Findings from ANOVA revealed that there were significant grade differences in school adjustment. A further univariate ANOVA analysis showed that the school adjustment of grade 7 adolescents was significantly higher than that of grades 8 and 9 (*P* = 0.000) (Table 1).

**Table 1:** Demographic information of the participants, their connections with NLEs, and their relationships with school adjustment. (*n* = 700)

**Variable**		**N (%)**	**NLEs (M** ± **SD)**	**F/t**	**School adjustment (M** ± **SD)**	**F/t**
Gender	Male	336(48.0)	2.36 ± 0.87	-1.241	3.98 ± 0.53	-2.393
	Female	364(52.0)	2.44 ± 0.78		4.07 ± 0.49	
Grade	Grade 7	238(34.0)	2.32 ± 0.80	2.381	4.14 ± 0.48	9.758^***^
	Grade 8	234(33.4)	2.43 ± 0.82		3.98 ± 0.52	
	Grade 9	228(32.6)	2.48 ± 0.85		3.96 ± 0.53	

^***^p < 0.001

Table 2 displays the intercorrelations between the factors evaluated for left-behind adolescents. NLEs were significantly and negatively associated with school adjustment (*r* =—0.305, *p* < 0.01), hope (*r* =—0.143, *p* < 0.01), and gratitude (*r* = -0.161, p < 0.01). School adjustment was significantly and positively related to hope (*r* = 0.407, *p* < 0.01) and gratitude (*r* = 0.458, *p* < 0.01). Based on the correlation matrix of the constructs, the following hypothesized model analyses were carried out. H_2_ was supported.

**Table 2:** Correlation among variables

No	Construct	1	2	3	4
1	SA	1			
2	NLE	− 0.305^**^	1		
3	HO	0.407^**^	-0.143^**^	1	
4	GR	0.458^**^	-0.161^**^	0.446^**^	1

*SA *School adjustment, *NLE* Negative life event, *HO* Hope, *GR *Gratitude. **p ≤ 0.01

### Mediation model with hope and gratitude

**Fig. 2:**
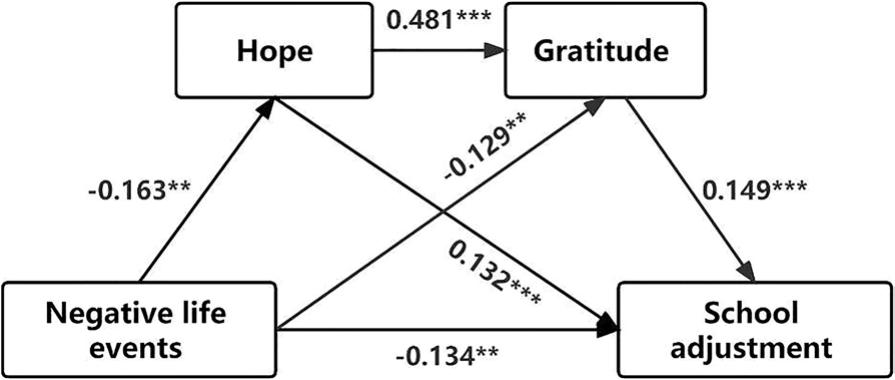
Path coefficient results. ^**^p ≤ 0.01, ^***^p ≤ 0.001

The SPSS plug-in Process Model 6 from Hayes [42] was applied. After controlling for the gender and grade of left-behind adolescents, NLEs were taken as an independent construct, school adjustment as the dependent variable, and hope and gratitude as serial mediating variables. Figure 2 displays the findings for the path coefficient.

The findings of multiple linear regression analysis of important study variables are illustrated in Table 3, that NLEs were found to significantly and negatively predict hope (β = -0.163, *p* < 0.001), gratitude (β = -0.129, *p* < 0.001), and school adjustment (β = -0.134, *p* < 0.001). Hope was a significant positive predictor of gratitude (β = 0.481, *p* < 0.001) and school adjustment (β = 0.132, *p* < 0.001), while gratitude was a significant positive predictor of school adjustment (β = 0.149, *p* < 0.001).

**Table 3:** The outcomes of regression analysis between variables

Items	Regression equation	Integral fitting index			Significance of regression coefficient	
**Outcome constructs**	**Predictive variable**	**R**	**R**^**2**^	**F**	**β**	**ε**
HO	Gender	0.164	0.027	6.379	-0.151	0.072(-2.103)
	Grade				-0.005	0.044(-0.115)
	NLE				-0.163	0.044(-3.708^**^)
GR	Gender	0.461	0.212	46.759	0.123	0.072(1.711)
	Grade				-0.010	0.044(-0.237)
	NLE				-0.129	0.044(-2.953^**^)
	Hope				0.481	0.038(12.788^***^)
SA	Gender	0.581	0.337	70.575	0.123	0.032(3.836^***^)
	Grade				-0.079	0.019(-4.043^***^)
	NLE				-0.134	0.020(-6.82^***^)
	Hope				0.132	0.019(7.085^***^)
	Gratitude				0.149	0.017(8.863^***^)

*SA *School adjustment, *NLE* Negative life event, *HO* Hope, *GR *Gratitude. ^**^p ≤ 0.01, ^***^p ≤ 0.001

Table 3 lists the evaluation of the mediating effect of hope and gratitude to examine the mediation approach. The Bootstrap test revealed that the 95% confidence intervals for the three paths did not contain 0, showing that the overall mediating effect was significant (effect = -0.053, 95% CI: -0.077, -0.030). Meanwhile, the total effect between NLEs and school adjustment was significant (effect = -0.134, 95% CI: -0.173, -0.095). NLEs affected the school adjustment of left-behind adolescents mainly through three intermediary paths: (1) NLEs → hope → school adjustment. The confidence interval of mediating effect did not contain a 0 value, indicating that the mediating effect of this path was significant (effect = -0.021, 95% CI: -0.035, -0.009), as it accounted for 11.23% of the total effect. (2) NLEs → gratitude → school adjustment. The confidence interval of mediating effect did not contain a 0 value, indicating that the mediating effect of this path was significant (effect = -0.019, 95% CI: -0.035, -0.006), as it accounted for 10.16% of the total effect. (3) NLEs → hope → gratitude → school adjustment. The confidence interval of mediating effect did not contain a 0 value, indicating that the mediating effect of this path was significant (effect = -0.012, 95% CI: -0.020, -0.005), as it accounted for 6% of the total effect. These findings suggested that hope and gratitude had a strong serial mediating influence on NLEs and school adjustment in adolescents. NLEs not only directly affected school adjustment but also indirectly affected school adjustment by affecting hope and gratitude. Therefore, the results were supported. This study further found that the implementation of hope and gratitude had a mediating effect between NLEs and school adjustment of middle school students, which verified H_2_, H_3_, and H_4_ (Table 4). The direct effect (effect size = -0.134) and total indirect effect (effect size = -0.053) accounted for 71.66% and 28.34% of the total effect (effect size = -0.187), respectively. Specifically, the indirect effect consists of three paths and accounts for 6%, 10.16%, and 11.23% of the total effects, respectively.

**Table 4:** The mediating effect of hope and gratitude

Items	Effect size	Boot SE	Boot CI		The proportion of effect size
			**Lower**	**Upper**	
Total effects	-0.187	0.022	-0.230	-0.143	100.00%
Direct effects	-0.134	0.020	-0.173	-0.095	71.66%
Total indirect effects	-0.053	0.012	-0.077	-0.030	28.34%
Indp1: NLE → HO → SA	-0.021	0.007	-0.035	-0.009	11.23%
Indp2: NLE → GR → SA	-0.019	0.007	-0.035	-0.006	10.16%
Indp3: NLE → HO → GR → SA	-0.012	0.004	-0.020	-0.005	6%

*Indp* Indirect path, *SA* School adjustment, *NLE* Negative life event, *HO *Hope, *GR* Gratitude, *CI* Confidence interval

Subsequently, post-hoc analysis is conducted to examine the primary influences of gender (or biological sex) and grade on hope and gratitude. The findings reveal that male left-behind children exhibit significantly higher levels of hope compared to their female counterparts. Moreover, hope levels were notably higher among students in grades 7 and 9 when compared to those in grade 8. Similarly, gratitude levels were also significantly elevated in grades 7 and 9 in comparison to grade 8 (Table 5).

**Table 5:** Gender, grade, and associations with negative life events and school

	**Gender**		**t**	**Grade**			**F**	**Post hoc test**
	**Male** **(*n* = 336)**	**Female** **(*n* = 364)**		**Grade 7 (*n* = 238)**	**Grade 8 (*n* = 234)**	**Grade 9 (*n* = 228)**		
	**M** ± **SD**	**M** ± **SD**		**M** ± **SD**	**M** ± **SD**	**M** ± **SD**		
**NLE**	2.36 ± 0.87	2.44 ± 0.78	-1.241	2.32 ± 0.80	2.43 ± 0.82	2.48 ± 0.85	2.381	-
**SA**	3.98 ± 0.53	4.07 ± 0.49	-2.393^*^	4.14 ± 0.48	3.98 ± 0.52	3.96 ± 0.53	9.758^***^	seven > eight, ninth
**HO**	3.35 ± 1.04	3.18 ± 0.87	2.249^*^	3.34 ± 0.90	3.14 ± 0.92	3.31 ± 1.04	3.154^*^	seven, ninth > eight
**GR**	5.12 ± 1.09	5.16 ± 1.02	-0.414	5.25 ± 1.01	4.97 ± 1.05	5.20 ± 1.08	4.813^**^	seven, ninth > eight

*NLE* Negative life event, *SA* School adjustment, *HO* Hope*, GR* Gratitude. *p < .05, **p < 0.01, ***p < 0.001

## Discussion

The current study investigated the link between NLEs and Chinese left-behind children’s school adjustment via the potential mediation of hope and gratitude. The results demonstrated that NLEs had a significant negative association with school adjustment, which verified H_1_, which is in accordance with prior research [45]. In other words, the more NLEs experienced by left-behind adolescents, the more possible they are to suffer from school maladjustment. Left-behind adolescents may have more negative events in their daily life. As a common stressor, NLEs are easy to cause pressure on them. Therefore, this may cause depression, anxiety, and other emotional problems and significantly affect the behavior of teenagers [46, 47], leading to the inability of individuals to adapt to the environment well. Too many stressful life events can easily lead to mental health problems [48], which may cause serious school adjustment problems [49].

These findings suggest that educators should be cautious about the possibility of NLEs. Programs that identify students' perceived traumatic life events should be encouraged to help them adjust more effectively. For instance, educators could create unique situations that give left-behind students a chance to adapt and the ability to handle difficult life difficulties. Schools may also offer relevant training programs in psychology and counseling services to release them from passive life experiences.

Hope acted as a mediator between NLEs and school adjustment, which verified H_2_. When left-behind adolescents faced more NLEs, a higher hope level would enable them to better adapt to school. This finding is comparable to a previous study, which found that hope could mediate individuals’ adjustment levels [25]. In school life, left-behind adolescents have a strong sense of hope regarding their ability to meet the more optimistic view of the current problem. According to Snyder [21], a person with a high level of hope has more pathway thinking and strong agency thinking. In addition, left-behind adolescents with a higher level of hope have more clear goals, and when confronted with setbacks, they can find multiple ways to solve problems and are more courageous to face challenges. Consequently, they become more proactive in coping with problems arising in adaptation, can solve problems from multiple angles, and have strong learning abilities and good academic performance. Furthermore, left-behind adolescents with a high level of hope may be cheerful and confident, so they have harmonious interpersonal relationships and can get along with their classmates. They have more positive emotions and high satisfaction. Based on this high level of hope, the adaptation level of left-behind adolescents has undoubtedly been improved. Therefore, in the process of education, parents and teachers should strengthen the training of students’ sense of hope, which is the ideal way to promote their good school adjustment and uphold the virtuous cycle of NLEs → high hope → positive coping → good school adjustment → higher hope.

Gratitude mediated the relationship between NLEs and school adjustment, which verified H_3_ that the effect of NLEs on left-behind adolescents partly directly affected their school adaptation and partly indirectly affected school adjustment through gratitude. This result is consistent with the research results of Bryant’s [31] study. Another research, which used an intervention on gratitude among college students, found that the intervention increased gratitude and adjustment [50].

According to Fredrickson’s [29] extended construction theory, gratitude, as a positive emotion, can improve the cognitive litheness of individuals, expand their thinking patterns, and improve the adaptability of individual coping styles. Persons with a high level of gratitude tend to focus on perceiving positive stimuli around them and get positive experiences, which may promote their school adjustment. As rural left-behind adolescents grow and become psychologically mature, they realize their parents’ hardships as migrant workers and as caretakers of their grandparents at home. The NLEs will make the left-behind adolescents have a negative emotional experience and reduce their ability to adapt to school development. Gratitude, as a positive psychological quality, can improve their ability to adapt to school. Therefore, attention should be emphasized on the training and education of future strategies to reduce and eliminate school maladjustment. Increasing the quality of gratitude will inspire, arouse, and give full play to its positive role in the school adjustment and development of rural left-behind adolescents. When left-behind adolescents have a high level of gratitude, it will help them better adapt to school life and learn and grow happily.

Hope and gratitude are the serial-mediated models of NLEs and school adjustment. Specifically, the NLEs are indirectly associated with school adjustment through hope and gratitude. When individuals experience too many NLEs, their self-adjusting and problem-solving abilities are affected, weakening their ability to express gratitude and leading to school maladjustment. According to the results of a six-year follow-up study on American college students, hope was a stable predictor of indicators of school adjustment such as academic achievement, high graduation rate, and low expulsion rate [51]. The study showed that the higher the level of gratitude, the more conducive to individual adjustment [52]. In other words, stressful events can increase the risk of school maladjustment. The influencing process is dynamic, and when favorable protective factors emerge, these factors can buffer the negative effect of NLEs on school adjustment. The positive psychology theory puts forward new ideas and perspectives for us to explain this phenomenon. As positive psychological qualities, hope and gratitude play an important role in rural left-behind adolescents’ social adaptation and development [53]. The traditional model of “disadvantage (left-behind) – stress – maladjustment” may be replaced to some extent by the model of “disadvantage (left-behind) – stress – positive psychological qualities (hope and gratitude) – well-adjusted”.

Furthermore, the post-hoc analysis conducted in this study sheds light on the influence of gender and grade on hope and gratitude among left-behind children. The finding that male left-behind children exhibit significantly higher levels of hope compared to their female counterparts is consistent with previous research on gender differences in coping mechanisms [54]. Boys' higher hope levels may indicate that they possess more effective strategies to deal with NLEs. This aligns with studies showing that hopeful individuals approach challenges with a positive outlook, believing in their ability to overcome difficulties and create a better future. Consequently, their enhanced hope may enable male participants to navigate through challenging life events more effectively, leading to improved school adjustment. For instance, a study by Landy et al. [55] found that male adolescents tend to engage in problem-focused coping strategies, which can contribute to their higher levels of hope and resilience.

The impact of grade on hope levels is also evident in the study's findings. Transitions to new educational environments, such as middle to high school, may play a crucial role in shaping hope levels differently in each grade. Seventh graders may experience heightened hope due to the excitement and anticipation of starting middle school, which represents a new chapter in their academic journey. Similarly, ninth graders' elevated hope might be fueled by the prospect of entering high school and encountering new opportunities for personal and academic growth. Previous experiences of successfully overcoming challenges and negative events may also contribute to higher hope levels in seventh and ninth graders [56]. These findings align with Fraser et al. [57], who noted that students transitioning to high school often perceive it as a fresh start, fostering hope and optimism. In contrast, the challenges associated with transitioning to a higher grade and the impending changes may explain the lower hope levels observed in eighth graders, making them more susceptible to the impact of negative life events on their school adjustment.

Furthermore, the study reveals a notable relationship between grade and gratitude levels among left-behind children. Seventh and ninth graders exhibit higher gratitude levels, indicating a greater inclination to appreciate positive aspects of their lives. This gratitude may act as a protective factor, buffering against the negative effects of life events on their school adjustment. Studies by Froh et al. [58] have highlighted the lower gratitude levels observed in eighth graders might stem from the challenges of adolescence and transitioning to a higher grade, potentially making them more susceptible to the impact of negative life events on their school adjustment.

In conclusion, the post-hoc analysis provides valuable insights into the relationship between gender, grade, hope, and gratitude among left-behind children. Understanding these associations can inform the development of targeted interventions to foster emotional well-being and school adjustment in this specific population. However, further research is necessary to explore the underlying mechanisms driving these relationships and to identify effective strategies to enhance hope and gratitude levels, particularly in critical transitional periods.

### Limitations and directions for future studies

The current study had some shortcomings that should be noted. The study’s cross-sectional design made it impossible to conclude causality and missed opportunities to monitor the school adjustment of left-behind adolescents in rural China. Additionally, the reliability of the findings might have been hampered by underreporting due to the use of measures based on participants’ self-reports. Despite these drawbacks, the current study examined the powerful mechanisms underlying the association between NLEs, hope, gratitude, and left-behind adolescents’ school adjustment, which have not been explored in the previous research literature.

## Conclusions

In conclusion, the findings showed a negative association between NLEs and school adjustment. Hope and gratitude were significantly more positively associated with school adjustment, whereas both were negatively associated with NLEs. Hope and gratitude mediated the effect of NLEs and school adjustment. These results support the recommendation for educators to create focused interventions to raise left-behind adolescents’ gratitude and hope. By doing this, these adolescents ought to be better able to handle difficult life circumstances and adjust to school. More prospective studies are required to confirm the dynamic association between NLEs, hope, gratitude, and school adjustment.

## Data Availability

The datasets generated and/or analyzed during the current study are not publicly available due to privacy and ethical restrictions but are available from the corresponding author upon reasonable request.

## Abbreviations

ANOVA: Analysis of Variance
ASLEC: Adolescent Self-Rating Life-Events Checklist
CHS: Children’s Hope Scale
CI: Confidence Interval
EFA: Exploratory Factor Analysis
GQ-6: The Gratitude Questionnaire-Six-Item Form
GR: Gratitude
HO: Hope
M: Mean
NLEs: Negative Life Events
SA: School Adjustment
SD: Standard Deviation
